# Knowledge and Practices Regarding Safe Household Cleaning and Disinfection for COVID-19 Prevention — United States, May 2020

**DOI:** 10.15585/mmwr.mm6923e2

**Published:** 2020-06-12

**Authors:** Radhika Gharpure, Candis M. Hunter, Amy H. Schnall, Catherine E. Barrett, Amy E. Kirby, Jasen Kunz, Kirsten Berling, Jeffrey W. Mercante, Jennifer L. Murphy, Amanda G. Garcia-Williams

**Affiliations:** ^1^COVID-19 Response Team, CDC; ^2^Epidemic Intelligence Service, CDC; ^3^Division of Environmental Health Science and Practice, National Center for Environmental Health, CDC.

*On June 5, 2020, this report was posted online as an *MMWR* Early Release.*

A recent report described a sharp increase in calls to poison centers related to exposures to cleaners and disinfectants since the onset of the coronavirus disease 2019 (COVID-19) pandemic (*1*). However, data describing cleaning and disinfection practices within household settings in the United States are limited, particularly concerning those practices intended to prevent transmission of SARS-CoV-2, the virus that causes COVID-19. To provide contextual and behavioral insight into the reported increase in poison center calls and to inform timely and relevant prevention strategies, an opt-in Internet panel survey of 502 U.S. adults was conducted in May 2020 to characterize knowledge and practices regarding household cleaning and disinfection during the COVID-19 pandemic. Knowledge gaps were identified in several areas, including safe preparation of cleaning and disinfectant solutions, use of recommended personal protective equipment when using cleaners and disinfectants, and safe storage of hand sanitizers, cleaners, and disinfectants. Thirty-nine percent of respondents reported engaging in nonrecommended high-risk practices with the intent of preventing SARS-CoV-2 transmission, such as washing food products with bleach, applying household cleaning or disinfectant products to bare skin, and intentionally inhaling or ingesting these products. Respondents who engaged in high-risk practices more frequently reported an adverse health effect that they believed was a result of using cleaners or disinfectants than did those who did not report engaging in these practices. Public messaging should continue to emphasize evidence-based, safe practices such as hand hygiene and recommended cleaning and disinfection of high-touch surfaces to prevent transmission of SARS-CoV-2 in household settings (*2*). Messaging should also emphasize avoidance of high-risk practices such as unsafe preparation of cleaning and disinfectant solutions, use of bleach on food products, application of household cleaning and disinfectant products to skin, and inhalation or ingestion of cleaners and disinfectants.

Survey questions were administered by Porter Novelli Public Services and ENGINE Insights on May 4, 2020, through PN View: 360,* a rapid turnaround survey that can be used to provide insights into knowledge and practices of targeted audiences. This opt-in Internet panel survey was administered to 502 U.S. adults aged ≥18 years using the Lucid platform (*3*); panel members who had not taken a survey in the previous 20 waves of survey administration were eligible to participate. Quota sampling and statistical weighting were employed to make the panel representative of the U.S. population by gender, age, region, race/ethnicity, and education. Respondents were informed that their answers were being used for market research and could refuse to answer any question at any time. No personally identifying information was included in the data file provided to CDC.^†^

Survey questions asked about general knowledge, attitudes, and practices related to use of household cleaners and disinfectants^§^ and about specific information regarding cleaning and disinfection strategies for prevention of SARS-CoV-2 transmission. Weighted response frequencies were calculated using SAS statistical software (version 9.4; SAS Institute). Because respondents were recruited from an opt-in panel rather than by probability sampling, no inferential statistical tests were performed.^¶^ Differences were noted when a difference of ≥5 percentage points was found between any estimates being compared.

The median age of respondents was 46 years (range = 18–86 years), and 52% of respondents were female. Overall, 63% of respondents were non-Hispanic white, 16% were Hispanic (any race), 12% were non-Hispanic black, and 8% were multiracial or of other race/ethnicity. Respondents represented all U.S. Census regions,** with 38% from the South, 24% from the West, 21% from the Midwest, and 18% from the Northeast.

Participants had limited knowledge of safe preparation of cleaning and disinfectant solutions (Figure 1). Overall, 23% responded that only room temperature water should be used for preparation of dilute bleach solutions, 35% that bleach should not be mixed with vinegar, and 58% that bleach should not be mixed with ammonia. In comparison, a higher percentage of respondents had knowledge about use of recommended personal protective equipment: 64% responded that eye protection was recommended for use of some cleaners and disinfectants, and 71% responded that gloves were recommended for use. Similarly, 68% responded that handwashing was recommended after using cleaners and disinfectants and 73% that adequate ventilation was recommended when using these products. Regarding safe storage of cleaners, disinfectants, and hand sanitizers, 79% of respondents said that cleaners and disinfectants should be kept out of the reach of children, and 54% that hand sanitizers should be kept out of the reach of children.

**FIGURE 1:**
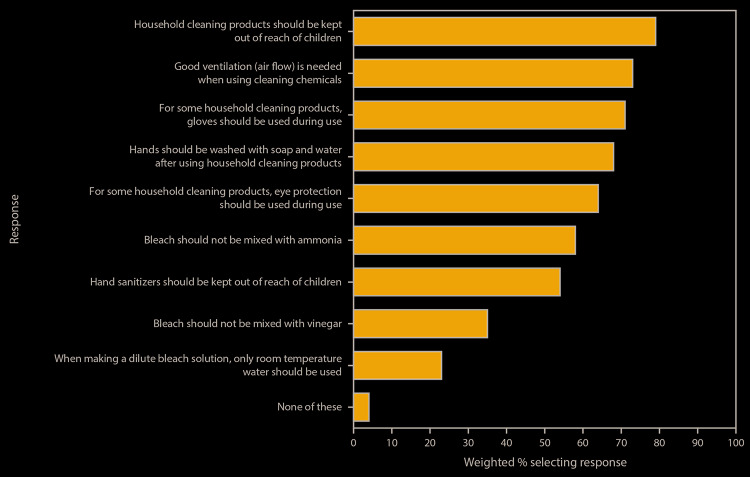
Knowledge about safe use of cleaners and disinfectants,*^,^^†^ based on responses to an opt-in Internet panel survey^§^ (N = 502 respondents) — United States, May 2020 * In response to the question ”Which of the following have you heard is true about using household cleaning products (such as bleach or Lysol)?”; response options reflected CDC recommendations for safe cleaning and disinfection. https://www.cdc.gov/coronavirus/2019-ncov/prevent-getting-sick/disinfecting-your-home.html. ^†^ In survey questions, the term “cleaning” referred to using a cleaner or disinfectant on surfaces or objects. Questions regarding storage of hand sanitizers were included with questions regarding storage of cleaners and disinfectants. ^§^ Survey administered by Porter Novelli Public Services through PN View: 360; respondents could select multiple responses to the question (all response options shown). Selection of the response “none of these” was exclusive (i.e., respondents could not select this response option in addition to other responses).

Respondents reported engaging in a range of practices during the previous month with the intent of preventing SARS-CoV-2 transmission (Figure 2). Sixty percent of respondents reported more frequent home cleaning or disinfection compared with that in preceding months. Thirty-nine percent reported intentionally engaging in at least one high-risk practice not recommended by CDC for prevention of SARS-CoV-2 transmission (*2*), including application of bleach to food items (e.g., fruits and vegetables) (19%); use of household cleaning and disinfectant products on hands or skin (18%); misting the body with a cleaning or disinfectant spray (10%); inhalation of vapors from household cleaners or disinfectants (6%); and drinking or gargling diluted bleach solutions, soapy water, and other cleaning and disinfectant solutions (4% each).

**FIGURE 2:**
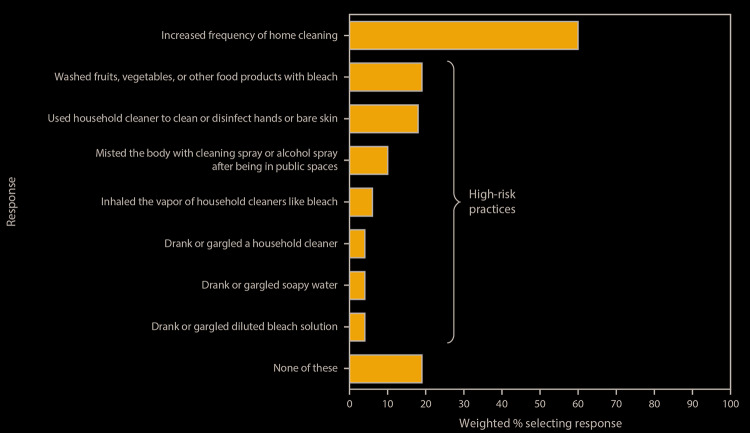
Cleaning and disinfection practices in the previous month with the intent of preventing SARS-CoV-2 infection,*^,^^†^ based on responses to an opt-in Internet panel survey^§^ (N = 502 respondents) — United States, May 2020 * In response to the question “In the past month, which of the following cleaning behaviors have you or a household member engaged in to prevent coronavirus?” ^†^ In survey questions, the term “cleaning” referred to using a cleaner or disinfectant on surfaces or objects. ^§^ Survey administered by Porter Novelli Public Services through PN View: 360; respondents could select multiple responses to the question (nine of 11 possible response options shown). Selection of the response “none of these” was exclusive (i.e., respondents could not select this response option in addition to other responses).

One quarter (25%) of respondents reported at least one adverse health effect during the previous month that they believed had resulted from using cleaners or disinfectants, including nose or sinus irritation (11%); skin irritation (8%); eye irritation (8%); dizziness, lightheadedness, or headache (8%); upset stomach or nausea (6%); or breathing problems (6%). Respondents who reported engaging in at least one high-risk practice more frequently reported an adverse health effect than did those who did not report engaging in such practices (39% versus 16%).

Approximately half (51%) of respondents strongly agreed and 31% somewhat agreed that they knew how to clean and disinfect their home safely. Similarly, 42% strongly agreed and 35% somewhat agreed that they knew how to clean and disinfect their home to prevent SARS-CoV-2 transmission. When asked who their most trusted sources of SARS-CoV-2-related cleaning and disinfection information were, the top three responses were CDC (65%), state or local health departments (49%), and doctors, nurses, or medical providers (48%).

## Discussion

This survey identified important knowledge gaps in the safe use of cleaners and disinfectants among U.S. adults; the largest gaps were found in knowledge about safe preparation of cleaning and disinfectant solutions and about storage of hand sanitizers out of the reach of children. Mixing of bleach solutions with vinegar or ammonia, as well as application of heat, can generate chlorine and chloramine gases that might result in severe lung tissue damage when inhaled (*4*,*5*). Furthermore, exposures of children to hand sanitizers, particularly via ingestion, can be associated with irritation of mucous membranes, gastrointestinal effects, and in severe cases, alcohol toxicity (*6*). The risk of ingestion and consequent toxicity from improperly stored hand sanitizers, cleaners, and disinfectants can also extend to pets (*7*).

Consistent with current guidance for daily cleaning and disinfection of frequently touched surfaces (*2*), a majority of respondents reported increased frequency of cleaning in the home. However, approximately one third reported engaging in high-risk practices such as washing food products with bleach, applying household cleaning and disinfectant products to bare skin, and intentionally inhaling or ingesting cleaners or disinfectants. These practices pose a risk of severe tissue damage and corrosive injury (*8*,*9*) and should be strictly avoided. Although adverse health effects reported by respondents could not be attributed to their engaging in high-risk practices, the association between these high-risk practices and reported adverse health effects indicates a need for public messaging regarding safe and effective cleaning and disinfection practices aimed at preventing SARS-CoV-2 transmission in households.

COVID-19 prevention messages should continue to emphasize evidence-based, safe practices such as frequent hand hygiene and frequent cleaning and disinfection of high-touch surfaces (*2*). These messages should include specific recommendations for the safe use of cleaners and disinfectants, including the importance of reading and following label instructions, using water at room temperature for dilution (unless otherwise stated on the label), avoiding mixing of chemical products, wearing skin protection and considering eye protection for potential splash hazards, ensuring adequate ventilation, and storing and using chemicals and hand sanitizers out of the reach of children and pets (*10*). Despite the knowledge gaps and high-risk practices identified in this survey, most respondents believed that they knew how to clean and disinfect their homes safely; thus, prevention messages should highlight identified gaps in knowledge about safe and effective practices and provide targeted information using innovative communication strategies (e.g., digital, social media) regarding safe cleaning and disinfection. These messages about cleaning and disinfection practices for COVID-19 prevention can be coordinated and disseminated through trusted sources of information such as national, state, and local public health agencies and medical providers.

The findings in this report are subject to at least four limitations. First, although survey responses were weighted to be nationally representative of U.S. demographics, whether responses among this opt-in panel sample are truly representative of knowledge, attitudes, and practices shared by the broader U.S. population is difficult to determine. Second, social desirability bias might have affected responses, with some respondents potentially overstating their perceived knowledge or underreporting engagement in high-risk practices; thus, these findings might underestimate the risk for exposures. Third, cross-sectional data captured in survey responses do not allow for direct attribution of specific outcomes, such as adverse health effects, to specific knowledge gaps or practices. Finally, responses were recorded at a single point in time and might not reflect ongoing shifts in public opinion or cleaning and disinfection practices by the public throughout the national COVID-19 response.

Efforts are ongoing to collect these data over time and to characterize knowledge gaps and practices among specific demographic and geographic groups. These data will allow for development and evaluation of further targeted messaging to ensure safe cleaning and disinfection practices in U.S. households during and after the COVID-19 pandemic.

**Summary**

**What is already known about this topic?**

Calls to poison centers regarding exposures to cleaners and disinfectants have increased since the onset of the COVID-19 pandemic.

**What is added by this report?**

An Internet panel survey identified gaps in knowledge about safe preparation, use, and storage of cleaners and disinfectants. Approximately one third of survey respondents engaged in nonrecommended high-risk practices with the intent of preventing SARS-CoV-2 transmission, including using bleach on food products, applying household cleaning and disinfectant products to skin, and inhaling or ingesting cleaners and disinfectants.

**What are the implications for public health practice?**

Public messaging should continue to emphasize evidence-based, safe cleaning and disinfection practices to prevent SARS-CoV-2 transmission in households, including hand hygiene and cleaning and disinfection of high-touch surfaces.

## Footnotes

* http://styles.porternovelli.com/pn-view-panels/.

† CDC obtained the survey data from Porter Novelli Public Services through a subscription license. Porter Novelli Public Services and its vendors are not subject to review by CDC’s Institutional Review Board; they adhere to professional standards and codes of conduct set forth by the Insights Association (https://www.insightsassociation.org/issues-policies/insights-association-code-standards-and-ethics-market-research-and-data-analytics-0).

§ Questions regarding storage of hand sanitizers were included with questions regarding storage of cleaners and disinfectants.

¶ https://www.aapor.org/AAPOR_Main/media/MainSiteFiles/NPS_TF_Report_Final_7_revised_FNL_6_22_13.pdf.

** https://www2.census.gov/geo/pdfs/maps-data/maps/reference/us_regdiv.pdf.

## References

[1] Chang A, Schnall AH, Law R, Cleaning and disinfectant chemical exposures and temporal associations with COVID-19—National Poison Data System, United States, January 1, 2020–March 31, 2020. MMWR Morb Mortal Wkly Rep 2020;69:496–8. 10.15585/mmwr.mm6916e1 32324720 PMC7188411

[2] CDC. Coronavirus disease 2019 (COVID-19): how to protect yourself & others. Atlanta, GA: US Department of Health and Human Services, CDC; 2020. https://www.cdc.gov/coronavirus/2019-ncov/prevent-getting-sick/prevention.html

[3] Coppock A, McClellan OA. Validating the demographic, political, psychological, and experimental results obtained from a new source of online survey respondents. Research & Politics 2019;6:1–14. 10.1177/2053168018822174

[4] Mrvos R, Dean BS, Krenzelok EP. Home exposures to chlorine/chloramine gas: review of 216 cases. South Med J 1993;86:654–7. 10.1097/00007611-199306000-00013 8506487

[5] National Center for Biotechnology Information. Compound summary: sodium hypochlorite. Bethesda, MD: National Library of Medicine, National Center for Biotechnology Information; 2020. https://pubchem.ncbi.nlm.nih.gov/compound/Sodium-hypochlorite

[6] Santos C, Kieszak S, Wang A, Law R, Schier J, Wolkin A. Reported adverse health effects in children from ingestion of alcohol-based hand sanitizers—United States, 2011–2014. MMWR Morb Mortal Wkly Rep 2017;66:223–6. 10.15585/mmwr.mm6608a5 28253227 PMC5657893

[7] Kore AM, Kiesche-Nesselrodt A. Toxicology of household cleaning products and disinfectants. Vet Clin North Am Small Anim Pract 1990;20:525–37. 10.1016/S0195-5616(90)50043-1 2180194

[8] Slaughter RJ, Watts M, Vale JA, Grieve JR, Schep LJ. The clinical toxicology of sodium hypochlorite. Clin Toxicol (Phila) 2019;57:303–11. 10.1080/15563650.2018.1543889 30689457

[9] Hall AH, Jacquemin D, Henny D, Mathieu L, Josset P, Meyer B. Corrosive substances ingestion: a review. Crit Rev Toxicol 2019;49:637–69. 10.1080/10408444.2019.1707773 32009535

[10] CDC. Coronavirus disease 2019 (COVID-19): cleaning and disinfecting your home. Atlanta, GA: US Department of Health and Human Services, CDC; 2020; https://www.cdc.gov/coronavirus/2019-ncov/prevent-getting-sick/disinfecting-your-home.html

